# Linking Childhood and Adult Criminality: Using a Life Course Framework to Examine Childhood Abuse and Neglect, Substance Use and Adult Partner Violence

**DOI:** 10.3390/ijerph10115470

**Published:** 2013-10-28

**Authors:** Anita Minh, Flora I. Matheson, Nihaya Daoud, Sarah Hamilton-Wright, Cheryl Pedersen, Heidi Borenstein, Patricia O’Campo

**Affiliations:** 1 Centre for Research on Inner City Health, The Keenan Research Centre in the Li Ka Shing Knowledge Institute, St. Michael’s Hospital, 30 Bond Street, Toronto, ON M5B 1W8, Canada; E-Mails: MathesonF@smh.ca (F.I.M.); Daoud@bgu.ac.il (N.D.); hamiltons@smh.ca (S.H.-W.); PedersenC@smh.ca (C.P.); BorensteinH@smh.ca (H.B.); O’CampoP@smh.ca (P.O.); 2 School of Population and Public Health, University of British Columbia, 2206 East Mall, Vancouver, BC V6T 1Z3, Canada; 3 Institute for Clinical Evaluative Sciences, 2075 Bayview Avenue, Toronto, ON M4N 3M5, Canada; 4 Dalla Lana School of Public Health, University of Toronto, 27 King’s College Cir, Toronto, ON M5S 1A1, Canada; 5 Department of Public Health, Faculty of Health Sciences, Ben-Gurion University, Beer Sheva 84015, Israel

**Keywords:** life course, intergenerational patterns, child abuse, substance use, criminality

## Abstract

Child abuse and neglect, considered criminal acts under the Criminal Code of Canada, play an important role in substance use, violence, and other criminal behaviour in adulthood. We adopted the life course perspective to identify modifiable contextual influences and co-occurring individual, social, and familial determinants associated with adult criminality. Using in-depth interview data, a sub-sample of 13 women who had recently experienced intimate partner violence, recounted their experiences of childhood abuse, their own substance use or criminality, as well as implications of these factors on their children’s life trajectories. For the purposes of this paper criminality was defined as child abuse and neglect, domestic violence, illegal substance use and underage alcohol use. Our objective was to explore, in our data: (1) patterns and trajectories of criminality from childhood to adulthood among women who were victims of violence, and (2) cumulative effects of early life exposures on experiences of criminality; with the aim of describing the life course perspective as a useful framework to understand criminality along the life trajectory. The analysis was not designed to demonstrate causal connections between early childhood and adulthood experiences of criminality. Rather we generated qualitative and quantitative hypotheses to guide future research in the field. Implications for research and interventions are discussed.

## 1. Introduction

The theory of intergenerational transmission of violence describes the tendency of exposure to violence or aggression in one generation to increase the likelihood of violence or aggression in the next generation. In its most common application the theory posits that children who are maltreated by their parents, guardians or other family members are, compared to those who grow up without abuse, more likely to grow up to maltreat their own children [1]. Crime theories would suggest that family disorganization and poor parenting practices result in weakened social bonds for the child and consequently low self-control [2,3,4,5]. Patterson specifically argued that antisocial behavior emerges from families marked by harsh discipline, negative parental interactions with their children and poor supervision [6]. Over the last three decades, there has also been growing support for the argument that there is an additional genetic basis to the transmission of personality traits related to criminality, such as aggression, anti-social behaviour, and impulsivity [7,8,9,10]. An early twin study by Christiansen [9], for example, demonstrated a stronger relationship between identical twins for violent crimes, suggesting a hereditary component to violence. More recent evidence suggests that though earlier research which partitioned genetic and social environmental transmission of such traits has been useful in helping to demonstrate the validity of both paradigms in the development of criminality, the reality is more complex [8,11]. The dynamic interplay between individual genotype and the environment during childhood is now thought to contribute to the development of criminal behaviour in adulthood. 

Child abuse is a criminal act under the Criminal Code of Canada [12]. Child neglect and physical/sexual abuse is a pre-cursor to serious social problems including adult victimization, sequelae of serious mental health problems and maladaptive parental practices, which can potentiate the intergenerational transmission of violence. Through social learning processes, children learn behaviours both from experiencing the ways in which others treat them, as well as observing how their own parents treat each other [13,14,15]. Exposure to interparental violence and resultant modeling may then teach children that violence is a means of resolving partner conflict, causing them to tolerate such behaviour and increase their likelihood of violence perpetration in adulthood [15,16,17]. In much of the literature on intimate partner violence (IPV) the theory of intergenerational transmission of violence is used to explain violence, both towards women by their partners, and by abused women towards their own children. For example, Heyman *et al.* found that mothers who experienced childhood victimization and who were exposed to interparental violence were more likely to perpetrate abuse towards their own children, and that this abuse in childhood was associated with partner violence perpetration in adulthood [18]. 

One framework that is particularly useful for considering early influences on adult experiences and outcomes is a life course perspective. A life course perspective in women’s and maternal and child health is increasingly being employed [19,20,21] while it is more developed within the criminality literature [4,5,22]. A life course perspective takes into account social, economic and environmental determinants of well-being with the goal of trying to better understand factors to ensure optimal health and developmental trajectories over a lifetime and across generations [20]. Several features of the life course approach help to create a framework by which we might systematically understand the determinants and consequences of child abuse or neglect, adult abuse, and intergenerational transmission of abuse.

First, a life course framework seeks to understand pathways and trajectories as an “integrated continuum of exposures, experiences and interactions” [20]. So, while research which explores the link between child and adult abuse without reference to a life course perspective does simultaneously consider these as variables in regression analyses, such approaches fail to uncover relevant modifiable contextual influences or co-occurring individual, familial and social determinants [23]. Second, cumulative impacts of adverse conditions early in life can determine adult development and well-being. Within these ideas of cumulative exposure and pathways and trajectories, a life course perspective seeks to further investigate the multi-level risk and protective factors (e.g., individual behaviours, family stressors or supports, neighbourhood deprivation or resources, and larger societal level policies) that shape current and future vulnerabilities, strengths and well-being. Thus, the life course framework seeks to capture the complexity and interaction of these early determinants in a dynamic fashion rather than just as indicators of the presence or absence of early risk factors on later outcomes. For example, Sampson and Laub [4,5], in their life course theory of crime, argue that while weakened social bonds can lead people on a trajectory to antisocial and criminal behavior; there are key turning or transition points along the life course (e.g., as employment, marriage and parenthood) that reestablish social bonds and promote prosocial behavior. Within their framework, movement away from adverse life experiences occurs when people are able to access positive human and social capital in the form of networks, social relationships and contexts [4,5,22,24]. Finally, a life course perspective enables us to link critical determinants over time using a developmental approach [25]. 

In addition, a life course framework helps to underline the importance of considering the contextual factors in which childhood abuse and exposure to violence occurs when drawing links between these events and adverse adult outcomes. In some ways, the connection between parental conflict and children’s adjustment occur because of the social and family contextual factors within which parental conflict arises, rather than as a direct result of parental conflict [26]. Much of the literature on this topic often fails to include contextual factors as possible mediators in explaining the intergenerational transmission of violence yet, as has been shown in the studies that do take these factors into account [16,26,27,28], they play quite a significant role.

We are therefore interested in looking at patterns and trajectories of criminality from childhood to adulthood among women victims of violence using a life course perspective. For this paper criminality is defined in several ways. First, from the perspective of the woman as a child, criminality takes the form of child abuse and neglect by her parents or guardians, which includes “acts of commission”, or omission by a parent or other caregiver that result in harm, potential for harm, or threat of harm to a child. The five principal forms of maltreatment are physical abuse, sexual abuse, physical neglect, emotional maltreatment and exposure to domestic violence [29]. The emotional, behavioural and psychological sequelae of child abuse and neglect affect childhood development and life course decisions that have implications for the woman and her children long after the experience of abuse.

From the perspective of the woman as an adult and her own children, criminality refers to illegal substance use (her own or her children’s). For children, illegal substance use might be drug-related or if underage alcohol-related as well. Criminality also refers to abuse and neglect of her own children. 

In using this lens to analyze our data, we will qualitatively explore whether and how the life course framework is useful to understand life trajectories involving criminality after experiences of child abuse. The paper will not demonstrate causal connections between early childhood and adulthood experiences of criminality, but will generate qualitative and quantitative hypotheses, from a life course perspective, that will guide future research in the field. 

## 2. Methods

The present study used data from the Housing and Women’s Health Study, a project conducted to examine the intersections between housing and health within women who had recently experienced intimate partner violence (IPV). This study was approved by the research ethics board at St. Michael’s Hospital in Toronto, Canada. Potential participants were recruited from five regions in Ontario, Canada. The regions were selected to include urban and non-urban locations as well as large, medium and small geographical areas. A network of relevant organizations, including women’s shelters, social housing authorities and community service organizations, were contacted to facilitate recruitment at their respective sites. Site representatives posted and distributed flyers to potential participants who were requested to telephone the study recruitment team to assess their eligibility to participate and schedule an interview. Eligibility criteria included women between the ages of 25 to 60 who resided in one of the three housing types (social, transitional or independent dwelling) and had experienced IPV within the past 5 years.

Semi-structured interviews were conducted with 41 women. The interviews focused on perceptions and experiences of housing stability/instability, health, and IPV. A subsample of 13 women voluntarily disclosed personal experiences related to child abuse, neglect, or substance use, and the abuse or neglect of their children. Interviews were conducted in the women’s own homes or in an agreed upon private location and lasted between 60 to 90 min. Interviews were audio recorded and transcribed verbatim. Following the interview, participants completed a short descriptive survey about their housing, health, and IPV experiences. A detailed description of our analytic process for this project has been published elsewhere [30]. 

A small number of women in the sample (n = 13), unprompted, recounted personal experiences of child abuse, neglect, or substance use, and the abuse or neglect of their children. The presence of such narratives within the interview data situate IPV, addiction, and other adversities, not only as circumstances faced in adulthood, but as symptoms of detrimental life trajectories set into motion early along the life course of these women. Drawing from the original analysis, the research team selected a number of relevant themes that pertained to childhood experiences of abuse or neglect to be further analyzed for this paper: childhood experiences, childhood abuse, children, young and youth, cycles, patterns of abuse, substance use and abuse, family influences, and mental health. The transcripts of the 13 women in the subsample were then re-analyzed in detail. 

### The Life Course Framework: Pathways and Cumulative Exposures

Two related key features of the life course framework guided the analysis: (1) cumulative advantages and disadvantages, and (2) mediating pathways linking variables from childhood to outcomes adulthood. The former emphasizes the accumulative nature of exposure to risk and resilience over time. Where one adverse or beneficial exposure tends to lead to another, over time, the risks a woman accrues during her life time will be analogous to a “chain of risks” [31]. Cumulative exposures may have “additive effects”, whereby each exposure increases risk for an individual; and, they may lead to a “trigger effect” wherein the final link along the chain of risks is the tipping point following which adverse outcomes immediately occur [18,32]. 

Pathways, meanwhile, refer to the multiple trajectories of education, work, and family that are followed by individuals and groups throughout society. In research and in theory, particular importance is placed on understanding the developmental implications of life transitions along the pathway, for e.g., transition from school to work, birth of first child, *etc.* [33]. Transitions early in life such as entry into adolescence can have long-term impacts because they influence subsequent transitions [4,5]. If, for example, transition into early adolescence involves criminality in the form of drug and alcohol use, school performance and work performance may suffer, resulting in a difficult transition from school to work. 

In our analysis, we were therefore interested in describing (1) cumulative exposures surrounding criminality as experienced by women throughout their life courses, and (2) the pathways linking a woman’s experiences with criminality in childhood to adulthood. For this analysis, quotes were selected for inclusion in this paper based on “representativeness” as assessed by two criteria: (1) the recurrence of a singular narrative across subjects or contexts, and (2) the ability of a narrative to “tell a story”, or otherwise comment on the interrelatedness of themes across a woman’s life course. All mention of places, names, and names have been removed to protect the identity of our participants.

## 3. Findings

### 3.1. Description of Subsample

Most of the 13 women (62%) lived in social housing, while the remainder lived in either transitional housing or independent dwellings. Almost two-thirds of the women (62%) were over 45 years old. Two-thirds (62%) had never been married, and of the remaining women 38% were separated or divorced. All of the women had children, and 92% had 2 children or fewer. All of the women were born in Canada, and 62% identified themselves as white (Caucasian). Sixty-nine percent of the women were on disability or not currently employed. Three-quarters of the women (77%) lived on annual household incomes of less than $20,000. The majority of the women (69%) had completed post-secondary education either at college or university.

During the interviews, each of the 13 women from our subsample described their history and experience with criminality, specifically abuse or neglect as a child, illegal substance use, and/or abuse or neglect of their own children. Ten of the 13 women reported experiencing all of the above throughout their lifecourse: child abuse or neglect as a child, substance use, and abuse or neglect of their own children. The remaining three women, either experienced abuse or neglect as a child, described abuse or neglect of their own children, or had a history of substance abuse.

### 3.2. Cumulative Exposures: Rooted in Context and Transmitted across Generations

#### 3.2.1. Contextual Features in the Chain of Risk

We first explored the use of the life course framework by applying the concept of the chain of risk, described in the section above, to women’s descriptions of the contextual features that characterized their experiences with criminality in childhood and adulthood. Poverty, housing instability, as well as chronic violence in the form of child abuse or IPV, and alcohol or substance use by parents, were all prominent contextual features of the childhood family environment described by women. Such contextual features were the backdrop against which women were exposed to a higher risk of criminality both in childhood and in adulthood. 

The family setting can impact life trajectories. For example, parental financial strain can set the stage for child neglect. One woman in the sample attributed her experience of her mother’s abuse and neglect and the family’s housing instability to inadequate finances. She subsequently experienced instability in her schooling environment. Another woman related familial alcoholism to her experiences of childhood abuse and neglect, setting the stage for housing instability.

*“…when I was born and raised, we moved every other month. We were evicted and I went to every school in [the town] at least three times. Three times in one year I was at the same school. It’s not a way to grow up, you know. I was born and raised on welfare and we just more or less fended each day to each day, you know?”* W24

*“Childhood was very chaotic. We were in and out of our house because my father’s violence and drinking and abuse. It was either with relatives or friends... And then my mom would leave. We would move to another city, and go back. There was a lot of movement. And then at 15 I finally left home and that took me on a road to living wherever I could find a place to live.”* W27

The cumulative nature of contextual risks associated with criminality also extended into adulthood. One woman described neglecting her son’s material needs when he was growing up because of her substance use. She further contextualized this period by describing her struggle with poverty and particularly their residence in social housing during her son’s childhood. As she describes, she felt that her son attributed her substance use to the cumulative effects of poverty during her adulthood. 

In the same quote, however, she describes poverty as an alterable factor along the “chain of risk”. She reflected that in his desire to break free from the cumulative effects of poverty on criminality, to break free from substance use, her son broke away from social housing in his adult life.

*“Okay, I think because of my housing, our housing situation when he was growing up because he saw me going from working you know, all this stuff to doing drugs and being totally unhealthy and always partying and not giving a shit about anything and not having too much food in the house you know what I mean like, I loved him with all my heart but it’s like you can tell somebody you love them but if you don’t show them it don’t mean a thing… I guess because he saw, he saw what I went through and that I guess he must have made up his…, kind of like when my mom and dad split up and that and I said to myself I will never be with an alcoholic, I will never be abused, I won’t whatever I think he probably did the same thing as far as the drugs go, it’s like I am going to break out of housing, I am going to get my own home, I don’t want to be poor, I don’t want to live in the box you know what, and he did that, he did that. Like he has a healthy lifestyle, they have their own home and all that stuff”* W07

Housing stability, or instability, was a recurring theme in our analysis of cumulative exposures. As mentioned above, housing instability, in the form frequent housing mobility, was a contextual feature of childhood for women and for their children. Residence in social housing, and in some cases homelessness, was another feature of housing instability that contextualized both childhood and adulthood.

“*It’s just*, *it’s amazing of survival*, *you know*, *for what you go through*. *Some of the things in my life where I have been*, *you know*, *dragged around with my mom*, *and I remember being with her and my mind was back that I had to have been before I was like five or six at least because well you didn’t go to kindergarten then so I didn’t go to school until I was six*. *But I remember times when my mom would take me and she would go hitchhiking*… *or just she would just drag me a long and that’s what she did she hitchhiked*. *You could do that a lot easier than you do now*. *But I remember sleeping in vacant houses*, *and you know and actually sleeping under a bridge one time and she just grabbed this cardboard to cover us up*. *So all that type of stuff I have lived through*” W71

In some cases, the cumulative nature of housing instability during women’s adulthoods began with the act of running away from the family home in childhood. As one woman describes, running away was a catalyst for instability in both housing and employment. While at the age of 15, she had already been exposed to criminality (in the form of childhood abuse by her father, and through her own substance use), her vulnerability towards criminality was enhanced by her early independence (she ran away) from her family and subsequent early housing and employment instability. She was also exposed to criminality in the form of substance use, within her intimate relationship with her male partner, with whom she was attached to at a young age.

“*I was already alcohol and drug addicted because of all of that. And I guess, you know, when I ran I just wanted out. I wanted a safe place to go because of my condition already. I just saw myself in one mess after the other. I used to work here and there. I married young because I was pregnant. My partner was drug addicted and an alcoholic too*.” W27

#### 3.2.2. Intergenerational Patterns as a Chain of Risk

The “chain of risk” can be applied to intergenerational patterns of criminality. Risk of criminality for a woman or her children can be enhanced through an accumulation of exposures across generations. Among the women in this study, criminality in many forms—child abuse and neglect, substance use, and intimate partner abuse—spanned the generations.

*“…when my parents split up and that and because I saw my dad beat up my mom so much when I was younger I swore I would never get in a relationship with an alcoholic and I would never stay with somebody that ever put a hand on me, never okay. So, it’s funny I think because of my background in my relationships I was always the aggressor, I was the one throwing things, getting angry, a slap occasionally if I felt like they were cheating on me or even if they did, they weren’t, if I just thought they were you know, I slapped a couple of guys a couple of times and got away with it you know?”* W07

*“…I also talked about the sexual abuse I went through as a child with my grandfather, the physical and mental and verbal abuse I went with my brother and because—and then I find out years later, like being an adult, my mother’s brother beat her.”* W29

Learned problem parenting, accumulated across generations, created environments of elevated risk for exposure to criminality as it pertained to childhood abuse or neglect. One woman’s exposure to criminality in childhood predisposed her to criminality in adulthood. Specifically, her experience of neglect by her mother during childhood was a contributing factor to her own neglect of her daughter.

*“…my way I do things, the way I grew up, I was portraying that. I was like sorta saying like, okay you’re four but you know you, I was four, and I was cooking my own breakfast…that was my mentality…But here’s [my daughter] who’s sick, who’s not feeling good and you know and I just didn’t have that compassion, so, there’s a few things that I kinda realized you know that I wasn’t the best for her keeping her.”* W52

### 3.3. Pathways: The Role of Family Environment and Coping along the Life Trajectory

The second aspect of the life course framework that we explored in the analysis of criminality was the concept of pathways and trajectories. We used women’s narratives to draw a pathway between the criminality present in their childhoods (*i.e.*, their experiences of childhood abuse and neglect), to the criminality experienced as adults (*i.e.*, their own substance use and abuse or neglect of their children). Here, we focused on mediating factors that connected women’s experiences with criminality in their childhoods to criminality in their adulthoods. We looked at women’s reflections about the various aspects of criminality that they felt were detrimental to their transitions from childhood and adolescence into adulthood. 

#### 3.3.1. Women’s Childhood Experiences of Abuse and Family Support

Having discussed the role of intergenerational transmission in the cumulative risk of criminality for women, it is unsurprising that for many women, the pathway to criminality began with their childhood family environment. Not every woman in our sample described their family as unsupportive, but most said that they lacked support from family members, and particularly parents. The absence of parental support was equated with a failure on the part of women’s parent(s) to provide protection against experiences of criminality in childhood, namely abuse, and to provide emotional resources that would have helped to buffer the negative emotional and psychological consequences of abuse.

*“[My father] is the one I should be mad at, but I am more mad at [my mother] because she knew. That’s why I’m mad. You knew and you didn’t stop it. You didn’t protect me somehow. I don’t know what—I don’t know. I know when I first did some of the counselling and brought it out, I still had a hard time with it, even saying it.”* W27

For one woman, an unsupportive parent-child relationship was the familial environment in which she experienced abuse in childhood as well as the reason that she could not disclose her experience with abuse.

*“…it was around my mom drinking…I was like molested and touched by my step dad but I couldn’t go to my mother and tell her that because she used to be so angry and she was always like screaming at me and blaming me for everything.”* W71

Another woman experienced a different example of a lack of parental support during childhood—being kicked out. Women’s descriptions highlighted the potential for a lack of parental support during childhood to have latent impacts during the life course, resulting in a variety of adverse experiences including mental health effects and criminality, in the case below, substance use

*“My mom never did groceries…I had a terrible childhood. A really abusive childhood. Kicked out at 15 years old. Yeah. Sometimes I ask myself why I turned out like this at middle forties, getting so messed up and confused over the drugs, because you think what happened being kicked out at 15 years old…”* W24

Support is important for emotional development. In the context of criminality within the family environment—one characterized by childhood abuse or neglect and a lack of support—the development of negative attitudes during childhood towards self was a recurrent theme. Attitudes such as low self-worth and feeling as if she “didn’t deserve better” were attributed, by many women, to a lack of protection and guidance from their parents during childhood.

*“I had an upbringing that was pretty horrible. And I’m not going to go into a bunch of detail about it. Um, well, my father was kind of a rotten bastard. My mother didn’t really protect us children, and, um, I, uh, had a really bad sense of self-worth.”* W47

*“I mean I sort of felt, not so much that I deserved it, but I don’t deserve any better than this you know, when your first male role model is your father, who beats you it’s hard to get out of that, even though I know it’s wrong, it doesn’t mean I change things.”* W09

The woman whose quote was the latter, later described her own children as having been neglected, regularly witnessing partner abuse between her and her partner.

#### 3.3.2. Parenthood and Women’s Children

As suggested above, all of the women in our sample became parents themselves—a significant stage in the life course of a woman. A number of these women reflected upon their children’s experiences of criminality as it pertained to abuse and neglect. In their interviews, a recurring theme was the desire of these women to provide their children with familial environments that contrasted with their own upbringing. Having lacked protection from abuse and neglect during childhood, women described wanting to give security, and protection to their children.

*“I loved [my son], I adored him especially when he was a baby and all that you know what I mean, I was always cuddling him like he was a little doll, and I was always so determined that there’s no way you would ever end up on the street or totally insecure like the way I did you know, without my mom and dad, and although I left his dad and that, like his dad would always take him every second weekend.”* W07

Even though women experienced criminality in the form of abuse during childhood and later tolerated abuse in adulthood, many women, when they became parents, expressed a need to protect their children from abuse. Protection over their children was expressed in the form of intolerance for violence towards their children, physical retaliation against their abusers, and for one woman, was the motivation for her to leave her abuser.

*“[My son] had an ash tray thrown at him. Not directly at him, but it hit the floor and slid into him and hit his foot. Of course, being a mom, you don’t hurt her children, right? Hurt me, don’t hurt my kids. And I went like a tiger at him. Of course I got my butt whipped in the end, but it was worth it. He knew never to go after my kid again.”* W29

*“And then [my partner] started getting physical with my kids…just the one time he got mad at my daughter, he went to go after her, I stepped in front of him to stop him, he grabbed me by the arms and threw me across the kitchen. I think I had 911 dialed before I hit the floor. And that was just the moment, okay this is it. I’m done.”* W65

For some women, the experience of childhood neglect included witnessing partner abuse between parental figures. Therefore, when they became parents themselves, these women recognized the negative contribution of a violent family environment to their children’s wellbeing. One woman, for example, talks about her continued participation in a violent relationship with her partner and its influence on her children’s wellbeing.

*“One of the worst things I think would be not seeing red flags for what they were and then kind of perpetuated a toxic relationship that was not beneficial to me or my children.”* W83

Finally, because women lacked support for mitigating the adverse impacts of abuse and neglect in their own childhoods, some women sought out institutionalized or formal support structures, such as counseling and therapy for their children. One woman, remembering her childhood experiences, described her motivation for seeking formalized support for her children.

*“I had to get counseling and the girls had counseling cause before I don’t think I realized, I thought oh they’re little…But then I’m remembering me, like, through my counseling to, but me as a child and my parents fighting, like I’ve never forgot that, right? So, so it was important for me to get them into counseling and me into counseling.”* W60

#### 3.3.3. Along the Pathway: Maladaptive Coping

Women recounted the ways they coped with exposure to criminal acts of childhood abuse and neglect. These became relevant to their transitions into adulthood and to subsequent criminality. Coping mechanisms varied in type, from attitudinal to behavioural, and resulted in a plethora of outcomes in adulthood, including mental illness, vulnerability to partner abuse, and substance use. Often, coping mechanisms were maladaptive. Furthermore, deleterious coping strategies that were employed in youth were, in the case of some women, continued into adulthood. 

For example, a number of women recounted having maladaptive coping strategies such as reluctance to disclose trauma and “suck it up” attitudes. These attitudes emerged from having to cope with the aspects of criminality faced in childhood, *i.e.*, their experiences of abuse, or the normalization of abuse in their childhood homes. Their attitudes and non-disclosing behaviors were later important contributors to their experiences of IPV in adulthood. Further, such attitudes were barriers for help—seeking at all stages in life. Women described the development of a “just suck it up” attitude and the non-disclosing behaviours in response to their childhood experiences:

*“I’d say it started when I was 15. I was raped at school. That’s the first time I had sex, I was raped at school…And I never told anybody till, I was 15, I never told anybody till I was about 30. I was too scared…I was confused by it. I was scared I didn’t know what to do. So I just suck it up and carry on.”* W65

*“I’ve seen my mother get beat to a pulp and hospitalized. I just always grew up thinking you’ve got to take the bad days with the good days. I didn’t know any different. So when things happened domestic in my home, that was my problem. I would have never ever dreamed of talking to anybody or thinking there was a way out or there was a place that I could go safe with my children.”* W24

Yet, for many women, the means of coping with abuse and neglect during childhood was to run away. Most of these women described running away from homes characterized by criminality in the family, including violence and child abuse. In childhood, the act of running away was motivated by a desire to leave unsafe and unsupportive homes in the hopes of finding a safer and more supportive environment. However, running away in childhood may have placed women on a pathway towards adverse experiences in adulthood, such as substance dependency, employment instability, and partner violence. One woman described running away when she was a child as a step towards her participation in a series of abusive partner relationships in her adulthood:

*“And one of my worst coping is to run to the next partner for comfort, but I find the same abusive, crazy characters. When all I want is comfort, right? And that’s been my pattern since I ran away [as a child]. I’m just looking for safe arms to run to and I don’t find them. I never find them. Yeah. That stuff is messing me up.”* W27

Some women also described using substances around this time of instability in their youth, or described their children using substances, as a means of coping with neglect and abuse in childhood. One woman described the self-medication that her son employed in response to the criminality that he was exposed to as a result of living with her.

*“…I hated that it got so tough with [my youngest son], with the place moving all the time. They are both violent like their father. I went to a bit of counselling to deal with him. When I found that he was using and in abuse, I was horrified. I know it’s not just the marriage but it’s my own personal life has not been stable, and he lived with me and he knows of the assaults and the rapes…”* W27

### 3.4. Jessica’s Story

To illustrate how pathways and cumulative exposures play out over a single life course, we have presented a case study of one woman as an example. For our purposes, we will call her Jessica. When we met Jessica, she had been living in social housing. At the time, she was older than the average age of our sample, with one adult child. She was on disability and made a total income of less than $20,000 per year and had a post-secondary education. Her story has been told in such a way that the interdependency of contextual and individual factors associated with criminality throughout her lifetime and across generations is highlighted. 

As a child, Jessica often witnessed her parents fighting. Her dad was abusive towards her mom. Jessica, herself, was exposed to criminality through her parents’ neglect and abuse. Her parents were separated when she was very young, after which, she and her siblings were placed into a number of orphanages and foster homes. She describes herself as a “trouble maker” at that time and eventually, in her teens, decided to quit school. She had already developed a pattern of running away as a coping mechanism at that point, having run away from the orphanage a few years earlier. She was homeless for a few years. She moved from home to home during this time, alternating between renting and living with family and friends. While homeless, she also went from job to job cleaning houses, and working at various retail stores. At one time when she was not employed, she stole food to eat. 

Having nowhere to call home, she lived for a brief time with her father. However, traumatic recollections of a past incident with sexual abuse by her father caused her to once again, run away. During this time, she further encountered incidents of sexual assault by two different people, both of whom were only acquaintances. Even though she describes her boyfriend during this time as someone that provided her with safety and security, her own insecurities, which stemmed from an abusive and neglectful family environment characterized by criminality, *i.e.*, childhood neglect and abuse, were a barrier for her involvement in the relationship.

*“I don’t know what it takes for somebody to believe that somebody really loves you but I was always very insecure I guess because my mom and dad split up and I never, nobody took me aside to explain to me why we were, and my brothers and sisters we were all stranded all over the place and um, so I had major issues with that.”*

For Jessica, exposure to criminality in childhood, having been abused and neglected, had an accumulative effect on her, which led her to run away. Running away was the trigger for a number of adverse conditions, such as homelessness, and employment instability, that would make Jessica vulnerable to further exposure to criminality. Moreover, Jessica’s negative feelings towards herself and distrust of others were barriers for her in experiencing support and security in a relationship.

Jessica eventually had a son when she became an adult. Unlike her own abusive experience within her family environment during childhood, she tried to protect her son from childhood abuse. When her son later had his own children, he was similarly non-abusive towards his children. She suggested that it was her affection towards her son that prevented him from being abusive. Her son, however, still struggled with alcoholism.

*“…he is a good dad um, and when he doesn’t drink and that like they’re fine, they have a good marriage, he’s not abusive okay, and he is very affectionate and that’s good because I was always affectionate with him and I was never abusive with him either you know, what I mean because I come from an abusive home so it’s like at least that’s one thing that I didn’t lay on him so today I am grateful for that because you know what he won’t even smack his kids.”*

In her later adulthood, Jessica began to use substances, including illicit drugs. Her son still lived with her at the time, still in his childhood, and she began to neglect him. The story of criminality for Jessica, particularly pertaining to substance use, continued across generations. It can be described, in part by her desire to differ from her father, who was an alcoholic. Meanwhile, her son expressed a need to differ from her, an illicit drug user, and choosing instead to use alcohol.

*“I always swore I would never be an alcoholic and I wasn’t, I never had a, I don’t have a problem with alcohol at all you know what I mean, I can take it or leave it and I think that’s because of my dad and my mom she never did drugs, she didn’t drink you know what I mean, my dad never did drugs so because I didn’t do the alcohol I ended up doing the drugs. Now my son he doesn’t smoke, doesn’t do no drugs but everybody, he does the alcohol you know, and when he gets drunk he gets into fights you know what I mean? Um, and he’s gotten into trouble a few times you know, stealing whatever when he was younger and that like that.”*

As an adult, Jessica eventually entered treatment for her substance abuse problem. She did so at the same time that she sought assistance to leave a violent relationship. She described that she must now be vigilant in her efforts to live a healthier lifestyle. She also reflected on the development of her ability to now recognize, as an adult, abuse as it influences her relationships and her own behaviour.

*“Today I understand that you can be emotionally abused, physically or mentally abused not just physically. There’s all kinds of abuse going on but back then I didn’t understand none of that. Today though I do, I understand it.”*

## 4. Discussion

The life course approach recognizes the complex and dynamic nature of criminality and its etiology. First, the approach embraces the multidimensionality of criminality because rather than attribute the cause to a single variable (the occurrence of childhood abuse, for instance), the life course approach integrates ecological, psychological, and economic perspectives into its treatment of crime etiology [34]. As such, in using a life course framework in looking at our qualitative data, we moved away from reductionism and towards a more holistic understanding of criminality. In the same vein, the life course approach considers the potential for a high-risk environment to exacerbate difficult behaviours such as substance use and aggression. Such behaviours may begin early in life [35], and have been demonstrated to have a genetic etiology [10,36,37]. By framing criminal behaviour as a product of intergenerational and environmental influences along a developmental trajectory, our study therefore acknowledges the importance of a gene-environment dynamic and contributes to an increasingly popular paradigm of thinking about criminality. Finally, the life course approach recognizes that the variables that influence criminality are not stable across time. That is, factors that influence criminal activity in childhood may not be the same as those that influence criminal activity in adulthood [34]. Our study therefore has implications for both qualitative and quantitative research in criminality that uses the life course approach. From a policy perspective, our exploration of the life course perspective in criminality may implicitly point to multiple opportunities for cessation of, or even prevention of criminality. 

With regards to the cumulative risk of criminality over the life course, our findings support the view that housing instability and poverty in childhood are contextual features that may contribute to a “chain of risks” [38,39]. The accumulation of negative contextual features has previously been found to trigger substance abuse/experiences of adult violent relationships [40,41]. Women in our sample experienced unstable housing in the family home during childhood, when running away, and later in adulthood. Moreover, in our sample of women who were predominantly low income, childhood poverty was the backdrop for experiences of criminality such as child abuse/neglect and for substance use. Women in our sample conceptually linked the cycle of substance use with the cycle of poverty, and further suggested that breaking out of social housing was one step in breaking away from criminality. 

Contextual factors seemed to be important in shaping risk and resilience. Disparate contextual features, such as financial strain, have previously been hypothesized to interact with family structure to magnify the risk of adverse child outcomes like social and emotional problems, and aggressive behaviours [42]. Our findings further point to poverty and neglect/abuse as examples of co-occurring adverse conditions of the family environment, which followed these women throughout the life course, influencing their risk of criminality. Future research might therefore continue to examine how the life trajectories of women who experience child abuse differ depending on contextual factors such as housing instability, or poverty. For example, because our sample was composed of mostly low-income women, future research might explore how the adult experiences of women who have experienced child abuse differ between women who did and did not also live in poverty/unstable housing. 

Our data also illustrated how neglectful parenting may also be transmitted intergenerationally, a finding consistent with previous studies [43,44,45]. The common assumption, with intergenerational patterns, has been that substance use and violence “run in families” that is that an individual’s risk of such behaviours increases if there is a similar parental history [1,32,46,47]. A life course approach suggests that it is more complicated. The life course concept of “linked lives” suggests that intergenerational influences from the life course of others may contribute to the explanation of behavioural patterns in any one individual [33]. The transmission of risks pertaining to criminality across generations may therefore be thought of as accumulative, or as a “chain of risk”. 

This was particularly evident around parenting practices and attitudes of adult victims. Women’s reflections around how they support and protect their own children reflected how their own experiences were shaped by their childhood experiences of being abused or of witnessing abuse and how they desired but rarely had the support of their own parents. While studies have shown that parental support is important to emotional development in childhood [25,48], our findings further highlight the impact of unsupportive family environments throughout women’s life courses. As a core tenet of Sampson and Laub’s sociogenic developmental theory of crime, they emphasized the mediating role of family and school social bonds in explaining criminality during childhood and in adolescence [4]. Social bonding for the women in our sample was poor during their childhood, due to a lack of support and the experience of abuse and neglect from family members. Furthermore, women who experienced considerable housing mobility or homelessness in childhood also experienced difficulty in forming lasting social bonds at school that would buffer them from criminality encountered in childhood. As a result, women in our sample experienced the far-reaching consequences of a lack of parental support (including reluctance to disclose trauma, feelings of poor self-worth, and difficulty creating new and supportive social networks due to distrust) that became influential for them along their pathways to parenthood. 

Women’s own parents were described as being not supportive because they did not adequately protect their children from abuse or did not provide the necessary resources that women needed to cope with the consequences of abuse in childhood. In contrast to their own upbringings, many women sought to provide their children with support and did so in the form of protection from abuse and parental presence. In some cases, formal supports such as counseling were seen as an important form of support during childhood and women who experienced IPV provided their children with such supports. During childhood, however, many women also experienced a normalization of abusive behaviours that went along with the development of negative attitudes towards self. A child’s risk of exposure to childhood abuse or neglect is higher when the parent exhibits tolerance towards abuse [1,15]. In addition, our data suggest that for women in our sample, such normalization of abuse, along with the negative attitudes towards self, carried over from childhood into adulthood, may be contributing factors to the inability of parents to protect their child from abuse or neglect. Future studies of families might therefore investigate what types of assistance would help women who experienced abuse to learn effective parenting practices and to ensure that child abuse or neglect is not repeated in the next generation. 

As mentioned, women’s attitudes towards self and relationships were negatively impacted by experiences with abuse and neglect during childhood. Specifically, childhood abuse and neglect co-occurred with the development of attitudes of low self-worth, and tolerance of violence within relationships. Both were described to coincide with the normalization of childhood abuse for women. Experiences with childhood abuse and neglect also led to the development of maladaptive coping strategies that persisted into adulthood Studies have found that coping strategies mediate the relationship between childhood experience with sexual abuse and adjustment in adulthood, and also have a role in determining dysfunctional behaviour in adulthood [49,50,51,52]. “Suck it up” attitudes and non-disclosure contribute to tolerance of violence within romantic relationships in adulthood and were barriers for help-seeking at all stages in life [1,53,54]. Our study further reveals that maladaptive coping strategies such as running away can place women on a pathway of greater vulnerability to criminality due to greater exposure to other contextual adversities such as housing and employment instability. Future research must therefore ask if and how modifications to the contextual features influencing the development of coping strategies during childhood would improve resilience and help seeking. Similarly, we need much more research examining the issue of resiliency such as why some women who experience criminality in childhood fare better when it comes to addiction, abusive relationship, and being non-abusive towards own children) than others. 

In closing our discussion of the study findings, we will recall and add a nuanced perspective to Sampson and Laub’s sociogenic developmental theory [4] which integrates life course theory with crime theory. Founded on the tenet that social bonding can mediate a pathway to crime, or to its reverse, conformity, Sampson and Laub, have implicated family and other social institutions of childhood e.g., school, *etc.* as sources for social bond formation. During the transition to adulthood, the quality of bonds formed in institutions that foster social control (e.g., employment and marriage), may influence whether an individual’s pathway to crime is redirected. In their study of men who had a history of crime, Sampson and Laub, found that those who experienced stable employment and good marriages refrained from crime in adulthood [4]. As mentioned above, women in our sample likewise experienced a destabilizing of social bonds at critical transition points across the life course. In childhood, women were poorly connected to their parents and to schools. In the transition to adulthood, women experienced unsuccessful transitions into stable housing, employment, and romantic partnerships. Our findings therefore similarly support the need for the formation of stable social bonds during the transition from childhood to adulthood. 

Though findings from women in our sample undoubtedly support Sampson and Laub’s theory, they also add nuance to the definition of “quality” of the turning point, particularly that there may be gender-specificity in such turning points. In particular, many women in our sample experienced increasing vulnerability in their romantic partnerships and marriages. So, for some women, these partnerships do not reflect a positive transition point but a continuation of the destabilization from childhood. Further, many women expressed the desire to become agents of stable social control in their own and in their childrens’ lives. Future research should explore gender differences in the type and quality of transition points between men and women along the pathway to crime and for women experiencing partner violence. Nonetheless, the importance of turning points in the study of criminality remains salient.

It must be noted that our findings are limited by the fact that we are reporting on information that was not systematically asked of all participants, rather, these themes and stories emerged unprompted in a subset of our sample. Thus, our findings cannot be generalized to all women in the study. Also, relating to the criminal behaviour of substance use, women often talked about this issue in the context of health and mental health, and housing instability, respectively, which were central focii of our interview. Finally, we only included in our sample, women who had experienced abuse as adults and disclosed that they had experienced abuse as children as well as criminal behaviour. Childhood abuse may be more prevalent among women in our sample and influencing their tendency to become involved in abusive relationships. We did not interview women who experienced childhood abuse and overcame the abuse cycle by staying free from violence as an adult and with their own children, as the study included only women who had experienced IPV. Ongoing efforts are needed to integrate our evolving understanding of criminality across the life course, and the multiple trajectories associated with risk and resilience.

## 5. Conclusions

Our study findings were presented in support of using the life course approach in future criminality research. It is only in doing so that research will be driven to identify multiple points of and types of family and personal interventions for victims of childhood abuse for the purpose of reducing drug use and intergenerational child abuse and neglect (e.g., intervention for children who leave home early, *etc.*); and, to develop effective training to screen for child abuse that would break the cycle of criminality and maladaptive coping. As Tomison [55] stated, evidence-based practice may be the means to the formation of a body of research from which a prevention strategy for criminality can be based. 

## References

[1] Renner L.M., Slack K.S. (2006). Intimate partner violence and child maltreatment: Understanding intra and intergenerational connections. Child Abuse Neglect.

[2] Gottfredson M.R., Hirschi T. (1990). A General Theory of Crime.

[3] Thornberry T.P., Krohn M.D., Farrington D.P. (2005). Applying interactional theory to the explanation of continuity and change in antisocial behavior. Integrated Developmental and Life-course Theories of Offending.

[4] Laub J.H., Sampson R.J. (1993). Turning points in the life course: Why change matters to the study of crime. Criminology.

[5] Sampson R.J., Laub J.H. (1995). Crime in the Making: Pathways and Turning Points Through Life.

[6] Patterson G.R., de Baryshe B.D., Ramsey E. (1989). A developmental perspective on antisocial behavior. Amer. Psychol..

[7] Farrington D.P., Wilson J.Q., Petersilia J. (2011). Families and crime. Crime and Public Policy.

[8] di Lalla L.F. (2002). Behavior genetics of aggression in children: Review and future directions. Develop. Rev..

[9] Christiansen K.O., Hood R. (1974). Seriousness of criminality and concordance among Danish twins. Crime, Criminology, and Public Policy.

[10] Schmidt L.A., Fox N.A., Rubin K.H., Hu S., Hamer D.H. (2002). Molecular genetics of shyness and aggression in preschoolers. Pers. Indiv. Differ..

[11] Karere G.M., Kinnally E.L., Sanchez J.N., Famula T.R., Lyons L.A., Capitanio J.P. (2009). What is an “adverse” environment? Interactions of rearing experiences and MAOA genotype in Rhesus monkeys. Biol. Psychiat..

[12] Government of Canada, Department of Justice http://www.justice.gc.ca/eng/cj-jp/fv-vf/laws-lois.html.

[13] Bandura A., McClelland D.C. (1977). Social Learning Theory.

[14] Franklin C., Kercher G. (2012). The intergenerational transmission of intimate partner violence: Differentiating correlates in a random community sample. J. Fam. Violence.

[15] Stith S.M., Rosen K.H., Middleton K.A., Busch A.L., Lundeberg K., Carlton R.P. (2000). The intergenerational transmission of spouse abuse: A meta-analysis. J. Marriage Fam..

[16] Ehrensaft M.K., Cohen P., Brown J., Smailes E., Chen H., Johnson J.G. (2003). Intergenerational transmission of partner violence: A 20-year prospective study. J. Consult. Clin. Psychol..

[17] Valdez C., Lim B., Lilly M. (2013). “It’s going to make the whole tower crooked”: Victimization trajectories in IPV. J. Fam. Violence.

[18] Heyman R.E., Slep A.M.S. (2002). Do child abuse and interparental violence lead to adulthood family violence?. J. Marriage Fam..

[19] Clark C., Caldwell T., Power C., Stansfeld S.A. (2010). Does the influence of childhood adversity on psychopathology persist across the lifecourse? A 45-year prospective epidemiologic study. Ann. Epidemiol..

[20] Fine A., Kotelchuck M. Rethinking MCH: The Life Course Model as an Organizing Framework. http://mchb.hrsa.gov/lifecourse/rethinkingmchlifecourse.pdf.

[21] Yoshihama M., Hammock A., Horrocks J. (2006). Intimate partner violence, welfare receipt, and health status of low-income african american women: A lifecourse analysis. Amer. J. Commun. Psychol..

[22] Carbone-Lopez K., Rennison C.M., Macmillan R. (2012). The transcendence of violence across relationships: New methods for understanding men’s and women’s experiences of intimate partner violence across the life course. J. Quant. Criminol..

[23] Mair C., Cunradi C.B., Todd M. (2012). Adverse childhood experiences and intimate partner violence: Testing psychosocial mediational pathways among couples. Ann. Epidemiol..

[24] Giordano P.C., Cernkovich S.A., Rudolph J.L. (2002). Gender, crime, and desistance: Toward a theory of cognitive transformation. Amer. J. Sociol..

[25] Bronfenbrenner U. (1986). Ecology of the family as a context for human development: Research perspectives. Develop. Psychol..

[26] Fergusson D.M., Horwood L.J. (1998). Exposure to interparental violence in childhood and psychosocial adjustment in young adulthood. Child Abuse Neglect.

[27] Banyard V.L., Williams L.M., Saunders B.E., Fitzgerald M.M. (2008). The complexity of trauma types in the lives of women in families referred for family violence: Multiple mediators of mental health. Amer. J. Orthopsychiat..

[28] Gómez A.M. (2011). Testing the cycle of violence hypothesis: Child abuse and adolescent dating violence as predictors of intimate partner violence in young adulthood. Youth Soc..

[29] Canadian Child Welfare Research Portal. http://cwrp.ca/child-abuse-neglect.

[30] Daoud N., O’Campo P., Pedersen C., Hamilton-Wright S., Minh A., Zhang Y.J., Matheson F.I. (2013). Housing stability/instability and health among women with past experiences of intimate partner violence. Violence Against Women.

[31] Ben-Shlomo Y., Kuh D. (2002). A life course approach to chronic disease epidemiology: Conceptual models, empirical challenges and interdisciplinary perspectives. Int. J. Epidemiol..

[32] Dube S.R., Anda R.F., Felitti V.J., Edwards V.J., Croft J.B. (2002). Adverse childhood experiences and personal alcohol abuse as an adult. Addict. Behav..

[33] Elder G.H. (1998). The life course as developmental theory. Child. Develop..

[34] Government of Ontario, Ministry of Children and Youth Services Integrated Life Course Theories.

[35] Moffitt T.E. (1993). Adolescence-limited and life-course-persistent antisocial behavior: A developmental taxonomy. Psychol. Rev..

[36] Kendler K.S., Prescott C.A., Myers J., Neale M.C. (2003). The Structure of Genetic and Environmental Risk Factors for Common Psychiatric and Substance Use Disorders in Men and Women. Arch. Gen. Psychiat..

[37] Verdejo-García A., Lawrence A.J., Clark L. (2008). Impulsivity as a vulnerability marker for substance-use disorders: Review of findings from high-risk research, problem gamblers and genetic association studies. Neurosci. Biobehav. Rev..

[38] Hawkins J.D., Catalano R.F., Morrison D.M., O’Donnell J., Abbott R.D., Day L.E., Seattle T., Development S., Mccord J., York N., Press G., Mccord J., Tremblay R.E. (1992). The seattle social development project: Effects of the first four years on protective factors and problem behaviors. Preventing Antisocial Behavior: Interventions from Birth through Adolescence.

[39] Burrow-Sanchez J.J. (2006). Understanding adolescent substance abuse: Prevalence, risk factors, and clinical implications. J. Couns. Develop..

[40] Stein J.A., Leslie M.B., Nyamathi A. (2002). Relative contributions of parent substance use and childhood maltreatment to chronic homelessness, depression, and substance abuse problems among homeless women: Mediating roles of self-esteem and abuse in adulthood. Child Abuse Neglect..

[41] Nair P., Schuler M.E., Black M.M., Kettinger L., Harrington D. (2003). Cumulative environmental risk in substance abusing women: Early intervention, parenting stress, child abuse potential and child development. Child Abuse Neglect.

[42] Wise S. (2003). Family Structure, Child Outcomes and Environmental Mediators: An Overview of the Development in Diverse Families Study.

[43] Robboy J., Anderson K.G. (2011). Intergenerational child abuse and coping. J. Interpers. Violence.

[44] Berlin L.J., Appleyard K., Dodge K.A. (2011). Intergenerational continuity in child maltreatment: Mediating mechanisms and implications for prevention. Child Develop..

[45] Lamela D., Figueiredo B. (2013). Parents’ physical victimization in childhood and current risk of child maltreatment: The mediator role of psychosomatic symptoms. J. Psychosom. Res..

[46] Dube S.R., Felitti V.J., Dong M., Chapman D.P., Giles W.H., Anda R.F. (2003). Childhood abuse, neglect, and household dysfunction and the risk of illicit drug use: The adverse childhood experiences study. Pediatrics.

[47] Whitfield C.L., Anda R.F., Dube S.R., Felitti V.J. (2003). Violent childhood experiences and the risk of intimate partner violence in adults Assessment in a large health maintenance organization. J. Interpers. Violence.

[48] Masten A.S., Coatsworth J.D. (1998). The development of competence in favorable and unfavorable environments: Lessons from research on successful children. Amer. Psychol..

[49] Guelzow J.W., Cornett P.F., Dougherty T.M. (2003). Child sexual abuse victims’ perception of paternal support as a significant predictor of coping style and global self-worth. J. Child Sex. Abus..

[50] Walsh K., Fortier M.A., di Lillo D. (2010). Adult coping with childhood sexual abuse: A theoretical and empirical review. Aggress. Violent Behavior.

[51] Frazier P., Tashiro T., Berman M., Steger M., Long J. (2004). Correlates of levels and patterns of positive life changes following sexual assault. J. Consult. Clin. Psychol..

[52] Merrill L.L., Guimond J.M., Thomsen C.J., Milner J.S. (2003). Child sexual abuse and number of sexual partners in young women: The role of abuse severity, coping style, and sexual functioning. J. Consult. Clin. Psychol..

[53] Bensley L., van Eenwyk J., Simmons K.W. (2003). Childhood family violence history and women’s risk for intimate partner violence and poor health. Amer. J. Prev. Med..

[54] Field C.A., Caetano R. (2003). Longitudinal model predicting partner violence among white, black, and Hispanic couples in the United States. Alcohol. Clin. Exp. Res..

[55] Tomison A.M. (2002). Preventing Child Abuse: Changes to Family Support in the 21st Century.

